# Surgical factors that contribute to tibial periprosthetic fracture after cementless Oxford Unicompartmental Knee Replacement: a finite element analysis

**DOI:** 10.3389/fbioe.2025.1543792

**Published:** 2025-04-04

**Authors:** Xiaoyi Min, Laurence Marks, Stephen Mellon, Takafumi Hiranaka, David Murray

**Affiliations:** ^1^ Nuffield Department of Orthopaedics, Rheumatology and Musculoskeletal Sciences, University of Oxford, Oxford, United Kingdom; ^2^ Department of Orthopaedic Surgery and Joint Surgery Centre, Takatsuki General Hospital, Osaka, Japan

**Keywords:** oxford unicompartmental knee, finite element analysis, periprosthetic tibial fracture, cementless fixation, fracture risk, surgical techniques

## Abstract

**Background:**

Tibial periprosthetic fracture (TPF) is a severe complication of cementless Oxford Unicompartmental Knee Replacement (OUKR) with patient risk factors including small tibial size and tibia vara with an overhanging medial tibial condyle. Surgical factors also influence fracture but remain poorly defined. This finite element (FE) analysis study identified surgical risk factors for TPF after OUKR and determined the optimal tibial component positioning to minimise fracture risk.

**Methods:**

Knees in two very high-risk, small, bilateral OUKR patients who had a TPF in one knee and a good result in the other were studied with FE analysis. Each patient’s unfractured tibia was used as a comparator to study surgical factors. The tibial geometries were segmented from the pre-operative CT scans and FE models were built with the tibial components implanted in their post-operative positions. The resections in the fractured and unfractured tibias were compared regarding their mediolateral position, distal-proximal position, internal-external rotation and varus-valgus orientation. Models of the TPF tibial resections in the contralateral sides were also built in both patients. The risk of TPF was assessed by examining the magnitude and location of the highest maximum principal stress.

**Results:**

In both patients, large differences were found in the position and orientation of the tibial components in the fractured and unfractured tibias with the components in the fractured tibias placed more medially and distally. Suboptimal saw cuts resulted in poor positioning of the tibial components and created very high local stresses in the bone, particularly anteriorly (157 MPa and 702 MPa in the fractured side vs. 49 MPa and 63 MPa in the unfractured side in patient 1 and 2 respectively), causing fractures.

**Conclusion:**

In small patients with marked tibia vara the surgery is unforgiving. To avoid fracture, the horizontal cut should be conservative, aiming for a 3 bearing, the vertical cut should abut the apex of the medial tibial spine, and extreme internal or external rotation should be avoided. The component should be aligned with the posterior cortex and should not overhang anteriorly. In addition, contrary to current recommendations, the tibial component should be placed in varus (about 5°).

## Introduction

Oxford Unicompartmental Knee Replacement (OUKR) is a treatment for end-stage isolated medial knee osteoarthritis (OA). The cementless version of OUKR was introduced in 2004 to resolve problems associated with cement, such as aseptic loosening, misinterpretation of physiological radiolucency and impingement (Mohammad et al., 2020a; Mohammad et al., 2021). It has demonstrated excellent functional outcomes with a 10-year survivorship of 97% (Campi et al., 2018). Periprosthetic fracture is an uncommon yet severe complication of cementless OUKR. Most cases are stress fractures in the tibia that occur during the early weight-bearing stage, although they can occur intra-operatively (Burger et al., 2022; Panzram et al., 2023). Depending on the fracture they can be treated conservatively, by internal fixation, or revision (Campi et al., 2018; Thoreau et al., 2021; Panzram et al., 2023).

The incidence of medial tibial periprosthetic fracture (TPF) varies in different series. For example, in the designer surgeons’ first 1000 cementless OUKR cases, no TPFs were reported (Mohammad et al., 2020b; Widari et al., 2024). Data from the National Joint Registry (NJR) for England, Wales, Northern Ireland and the Isle of Man shows that fractures around the knee do occur with the incidence in the first year being less than 0.5% (Mohammad et al., 2020a). However, these are not all fractures at the medial proximal tibia. Although there are more revisions for fracture with cementless than cemented, their incidences of fracture are similar (Burger et al., 2022). This suggests that the proportion of cementless fracture cases treated by revisions is higher than the proportion of cemented cases.

A much higher TPF incidence has been reported in studies carried out in Japanese cohorts (3.8%–8%) (Hiranaka et al., 2020; Yoshikawa et al., 2020; Kamenaga et al., 2021). The main cause of this is the difference in tibial morphology between races: Asian populations generally have smaller tibias, more overhanging medial tibial plateaus, and more tibia vara, with bowing of the tibia and varus inclination of the tibial plateau (Nagamine et al., 2000; Tang et al., 2000; Yau et al., 2007), which are considered risk factors for TPF (Burger et al., 2022).

**FIGURE 1:**
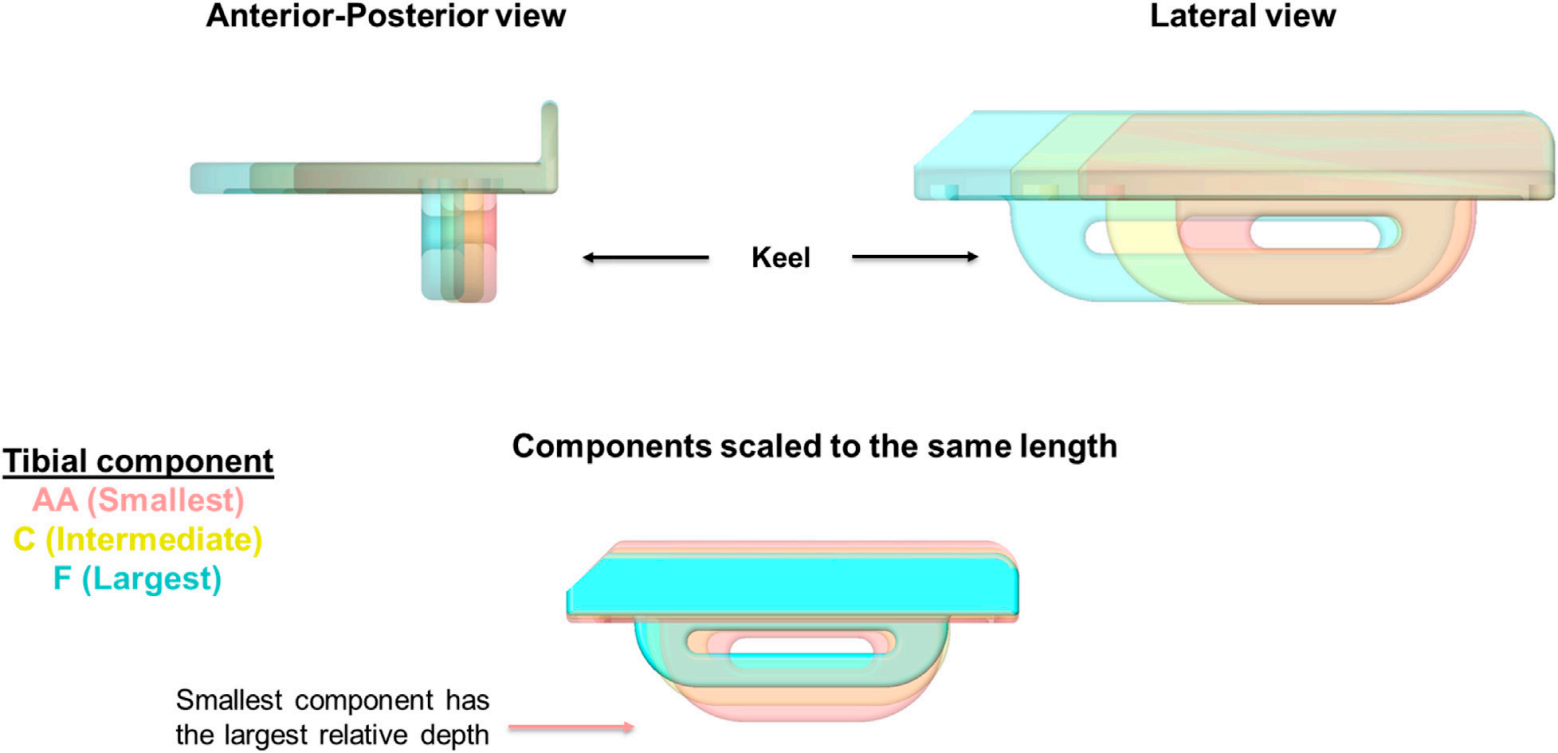
OUKR tibial components of Size AA, Size C, and Size F. When the components are scaled to have the same length, the smallest component (Size AA) has the largest depth, implying the depths of smaller components are relatively larger. Figure adapted from (Rahman, 2022).

The vertical protrusion beneath the tibial tray called the “keel” acts as the fixation component in OUKR tibial components (Figure 1). The fixation of a cemented tibial component is achieved by filling the gap between the keel and the slightly oversized keel slot with bone cement. The fixation of a cementless tibial component is achieved through the interference fit of the porous, hydroxyapatite-coated keel. Hiranaka et al. (2020) reported that patients having small size (Size AA or Size A) tibial components, which are commonly used in Asia (Wang et al., 2021), showed a higher risk of TPF after cementless OUKR, which was also found in a retrospective radiographic study (Watrinet et al., 2024). In the current OUKR design, regardless of their size (from Size AA to Size F), all tibial components have a keel which has the same depth and width. This means that keel slot cuts are deeper relative to the size of the tibia in tibias using smaller components (Figure 1). Relatively more bone is removed in these tibias leaving less bony support beneath the tibial component. With excessive tibial resection being one of the risk factors for TPF after cementless OUKR (Burger et al., 2022), this probably contributes to the higher risk of TPF found in the Asian population.

A previous systematic review has summarised the risk factors for TPF after UKR and categorised them into patient-related risk factors or surgical risk factors (Burger et al., 2022). Patient-related risk factors are high BMI, female gender, poor bone quality (e.g., osteoporosis), small tibial size and overhanging tibial plateau (Burger et al., 2022). A Medial Eminence Line (MEL) was introduced to assess the risk of TPF after cementless OUKR based on tibia morphology from pre-operative anteroposterior (AP) radiographs (Yoshikawa et al., 2020). The MEL starts from the apex of the medial tibial spine and extends parallel to the tibia anatomical axis. Based on the position of the MEL line relative to the medial cortex, tibias can be categorised into extramedullary type, where the MEL penetrates the medial cortex, and intramedullary type, where the MEL does not penetrate the medial cortex. Extramedullary-type tibias have a higher risk of TPF after cementless OUKR (Yoshikawa et al., 2020). The surgical risk factors that were identified are excessive tibial resection and component over- or under-sizing (Burger et al., 2022) and there are likely to be other surgical risk factors. Kamenaga, Hiranaka (Kamenaga et al., 2021) showed that patients who experienced TPF after cementless OUKR generally had a shorter distance between the keel and the posterior cortical bone compared to those without fracture. Placement of the tibial component too medial or too distal shortens this posterior keel-cortex distance (KCD) and subsequently increases the risk of fracture (Kamenaga et al., 2021). Kamenaga, Hiranaka (Kamenaga et al., 2024) further suggested that varus implantation of the tibial component could increase KCD and potentially lower the risk of TPF, especially in patients with overhanging medial tibial plateaus.

Finite element (FE) analysis is a widely used computational tool in orthopaedics to understand bone fracture (Keyak et al., 1990; Pegg et al., 2020; Stoddart et al., 2022; Stoddart et al., 2024). It enables the visualisation of stress and strain in the host bone during implantation and load bearing. It is therefore possible to explore in detail surgical factors that might influence the risk of fracture. Due to the complex microstructure and inhomogeneous nature of bone, analysis of crack propagation in fracture is complicated and computationally demanding. Maximum principal stress or strain has been widely used as a surrogate measure to indicate where fracture would initiate (Schileo et al., 2008; Ota et al., 1999; Bessho et al., 2007; Koivumaki et al., 2012; Stoddart et al., 2022).

The study aimed to identify, using FE analysis, surgical risk factors for TPF after OUKR and to determine the optimal position of the tibial component to minimise the risk of fracture. To exclude the influence of patient risk factors, both knees in two high-risk patients with bilateral OUKR who had a TPF in one knee and a good result in the other were studied. Each patient’s unfractured tibia was used as a comparator to study various surgical risk factors associated with TPF.

## Materials and methods

### Knees studied

**FIGURE 2:**
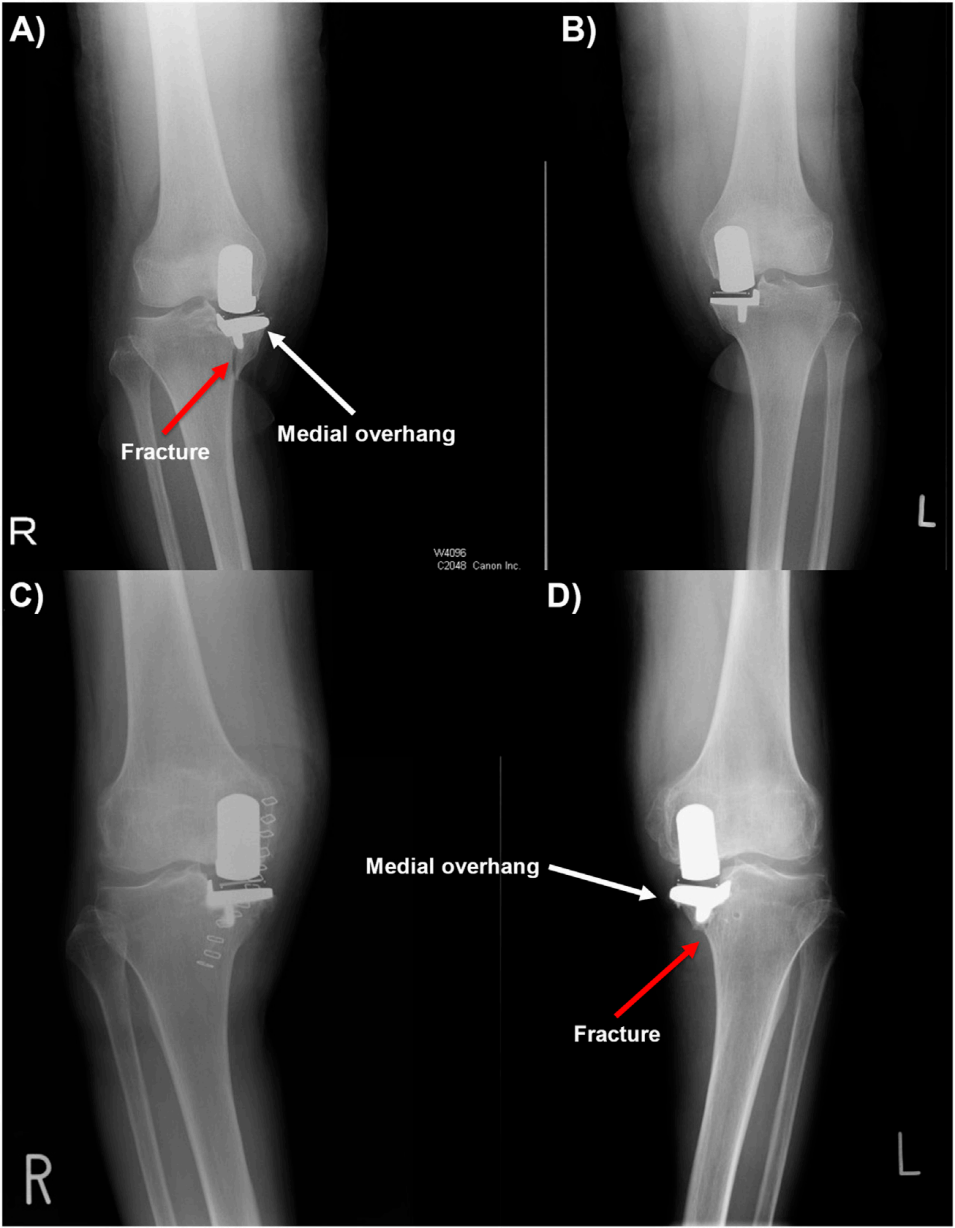
**(A, B)** Post-operative anteroposterior radiographs of patient 1, showing periprosthetic fracture in the right tibia. **(C, D)** Post-operative anteroposterior radiographs of patient 2, showing periprosthetic fracture in the left tibia with tibial component medial overhang.

Two high-risk patients who had bilateral medial OUKR and sustained a TPF in one knee were identified. Both patients were female and had extramedullary tibias identified with MEL and tibia vara. As these two fractures came from one knee in two patients who had received bilateral OUKR, the patient risk factors of high BMI or female gender can be ruled out. The same-sized tibial components were used in both the fractured and unfractured knees. Patient 1 received a Size C cementless tibial component in both knees while only the right tibia fractured (Figures 2A, B). Patient 2 received a Size A cemented tibial component in the left tibia which fractured, and received a Size A cementless component in the other tibia (Figures 2C, D). The patients had had pre-operative, post-operative and post-fracture AP and lateral radiographs, and pre-operative, and post-fracture computed tomography (CT) scans, allowing FE models with correctly positioned tibial components to be constructed.

The geometries of the four tibias of patient 1 and patient 2 were segmented from the pre-operative CT scans with MIMICS (Materialise, Leuven, Belgium). The CT scans were recorded in the format of Digital Imaging and Communications in Medicine (DICOM). They were taken with a slice thickness of 2 mm and the pixel spacing was (0.781, 0.781) in the transverse plane.

### Assessment of patient-related risk factors

Bone quality, tibial size and morphology of the tibial plateau were assessed in the fractured and unfractured tibia. Bone quality was assessed by the comparison of the elastic modulus of the trabecular and cortical bone of the fractured and unfractured tibias. As subchondral sclerosis which is a symptom of OA causes the bone to densify and may lead to a change in response to stress (Chen et al., 2013), the presence of sclerotic bone was also assessed from the pre-operative CT scans. The size and shape of the fractured and unfractured tibias were compared in terms of the aspect ratio of the tibial plateaus. The morphology of the tibial plateaus was compared by measuring their medial proximal tibial angle (MPTA), which is the medial angle between the tangent line of the medial and lateral tibial plateau and the tibial mechanical axis. The MELs were drawn on the pre-operative AP radiographs to identify if the tibias were extramedullary type or intramedullary type.

### Assessment of surgical risk factors

The geometries of the four tibias of patient 1 and patient 2 segmented from the pre-operative CT scans with MIMICS (Materialise, Leuven, Belgium) were imported into Abaqus 2020 (Dassault Syste`mes, France) for FE analysis. A previous study demonstrated that a shortened tibia would not affect the outcomes (Simpson et al., 2009). Also, truncating the tibia to the current length did not create a stress concentration at the bottom face of the shortened tibia, affecting the stress near the tibial resection. Thus, only the proximal tibias were modelled for analysis efficiency. The positions of the tibial components were reconstructed in 3 dimensions in SolidWorks 2022 (Dassault Systèmes, France) referring to the post-operative AP and lateral radiographs. The component was adjusted to a position where the AP and lateral flattened image of the 3-dimensional geometries best fit the radiographs. The positions of the vertical and horizontal cuts were found correspondingly assuming the cuts were perfectly orthogonal to each other with no gaps between the component and the cut surfaces. The difference in the positions of the pair of components in each patient was measured in terms of their mediolateral position, proximal-distal position, internal-external rotation and varus-valgus orientation. The tibial resections were made in the FE model with the revolve cut feature and the keel slots were created with the Boolean function in Abaqus using a Python code adapted from a previous study (Pegg et al., 2020).

In patient 1, when the keel was placed in the middle of the keel slot in the AP direction as the surgical technique suggested, the keel slot cut through the bone and created a hole in the anterior cortex. The damage to the cortex would greatly reduce the load-bearing ability of the bone. Therefore, a second scenario in which the keel slot was shifted backwards by 1.81 mm was also modelled. In this scenario, no hole was created in the tibia and the keel was closer to the front of the keel slot than to the back. This was intended to provide a more conservative estimate of the maximum stress in the fractured tibia when a hole, which would act as a stress raiser and a more direct cause of fracture, is not presented.

As the tibial components were overhanging medially and anteriorly in the fractured tibias of both patient 1 and patient 2, components one size smaller (Size B for patient 1 and Size AA for patient 2) were also tested in the models. The same horizontal and vertical tibial resections were made, and the position and size of the keel slots were adjusted accordingly.

#### Mesh

The tibias were modelled as deformable parts and were meshed with 2.4 mm quadratic tetrahedral elements (C3D10M). The mesh was refined to 0.5 mm in the fractured tibia of patient 1 and was refined to 0.6 mm in the other three tibias based on the results of a mesh convergence study: the percentage changes in the highest maximum stress in the tibias should be less than 5% when elements 0.1 mm smaller were used. The percentage changes in the highest maximum principal stress in the tibia were 2% in the fractured tibia of patient 1 when 0.4 mm elements were used. Thus, mesh refinement with elements of 0.5 mm was applied at the keel slot. Similarly, the results converged from a mesh size of 0.6 mm in the other tibias. Thus, 0.6 mm elements were used to save analysis time.

As the tibial components made from cobalt chromium molybdenum alloy were orders of magnitude stiffer than trabecular bone, they were modelled as rigid and meshed with 2.4 mm quadrilateral rigid elements (R3D4). The mesh on the keel was refined to 0.8 mm to better capture the curvature at the quadrant shape anterior and posterior margins.

#### Contact

The width of the keel slot was 0.16 mm narrower than that of the keel to simulate an interference fit contact (MacAulay et al., 2024). The keel was placed in the middle of the keel slot in the mediolateral direction, and this led to the keel elements penetrating those of the tibia at the contact interface. At the start of the analysis, an interference fit algorithm was defined to resolve this initial overclosure between the keel and the bone: the bone elements were gradually displaced until there was no more penetration. The contact between the bone and the tibial component was frictionless in the interference step, and then a friction coefficient of 0.99 was applied to model the contact between the porous keel and the trabecular bone in the loading step (Min et al., 2024). Using the current keel-cut saw blade, the keel slot would be 2 mm offset from the keel in the sagittal plane. Thus, no other keel surfaces were in contact with the keel slot.

#### Boundary conditions and loads

The distal ends of the proximal tibias were constrained in all six degrees of freedom throughout the analysis. A 1,000 N force was applied to the tibial component perpendicular to its superior surface to simulate the load transfer in the medial compartment during gait (Armillotta et al., 2023). The direction of the load stayed perpendicular to the tray. The loading position represented the position of the bearing when it was 1 mm away from the tibial wall and 8 mm posterior to the middle line of the tray. This simulated the mediolateral position and the posterior-most position of the bearing during step-up motions (Pegg et al., 2016) and it tends to occur in knee flexion, e.g., stair-climbing, when the knee joint loads are high (Kutzner et al., 2010). The tibial component was fully constrained in all degrees of freedom in the interference step and was free to move in the loading step.

#### Material properties

The elastic modulus and Poisson ratio of the bone were assigned to the proximal tibia in the FE models using the py_bonemat_abaqus python package (Pegg and Gill, 2016). This software tool reads Hounsfield Units (HU) from the pre-operative CT scans and converts them into Young’s modulus of the bone elements. This modelled the bone as a heterogeneous linear elastic material with a Poisson ratio of 0.35.

### FE analysis of fractured tibial resections in the unfractured tibias

The tibial resections in the two fractured tibias in patient 1 and patient 2 were replicated in the opposite (unfractured) tibias. A 1,000 N force perpendicular to the superior surface of the tibial component was applied to the same loading position: the position of the bearing when it was 1 mm away from the wall and 8 mm posterior to the middle line. All other settings in the model also remained the same.

### Assessment of the effects of component’s position and orientation on the risk of fracture

The effects of the tibial component’s mediolateral position, proximal-distal position, varus-valgus orientation and internal-external rotation on the risk of fracture were assessed in terms of the highest maximum principal stress in the bone. Bone was found to behave more like a brittle material than a ductile material (Bessho et al., 2007) and the maximum tensile principal stress is generally used to assess brittle failure (Moss et al., 2013). Hence, maximum principal stress, the maximum normal stress on a material when it is under loads, was used to indicate the fracture risk in this study.

The tibial resection (vertical cut and horizontal cut) in the two unfractured tibias of patient 1 and patient 2 were moved medially by 1 mm, 3 mm and 5 mm, laterally by 1 mm, 3 mm and 5 mm, distally by 1 mm, 3 mm, and 5 mm, or proximally by 1 mm, 3 mm and 5 mm from their original positions (hereafter called reference positions). If the change in position resulted in the keel slot cutting through the tibial bone, a movement 1 mm smaller was tested instead. The cuts were also rotated by 5°, 10° and 15° internally, externally, into varus or valgus. The internal-external rotation was made about the midpoint of the AP cut. The varus-valgus rotation was made so that the vertical cut sits right next to the medial tibial spine.

A 1,000 N vertical load was applied to the superior surface of the tibial tray in each model. It was assumed that the corresponding placement of the femoral component and bearing thickness choice would be made to ensure that the bearing was placed at an optimal position. This bearing placement should not lead to bearing impingement onto the vertical tibial wall throughout the full extension-flexion motion. Thus, the same loading position was kept. Other settings and parameter inputs in the model all remained the same.

The 3-dimensional models of the tibias with the tibial resection and keel slot created were imported into SolidWorks 2022 (Dassault Systèmes, France). The shortest distance between the keel slot and the surfaces of the tibia, either posterior or anterior, was measured in each model and this measurement will be referred to as the slot-cortex distance (SCD) from this point forward.

## Results

### Comparison of patient-related risk factors

No large difference was found in the bone quality, tibia size and medial condyle morphology between the fractured and unfractured tibias in either patient 1 or patient 2. Both pairs of tibias showed trabecular bone and cortical bone of similar elastic modulus, and the aspect ratio of the tibial plateaus was similar in each patient (Table 1). In the pre-operative CT scans, all four tibias in these two patients showed sclerosis at the medial tibial plateau. In patient 1, the MPTA was 80 ° in both the fractured and unfractured tibia, and in patient 2, the MPTA was 79 ° in both tibias. All four tibias were extramedullary type with the MEL penetrating the medial cortex on the pre-operative AP radiographs.

**TABLE 1:** Comparison of the bone elastic modulus and the size of the tibial plateau of the fractured and unfractured tibias in patient 1 and patient 2.

Patient 1	Unfractured (Left)	Fractured (Right)
Trabecular bone elastic modulus	∼200–2,000 MPa	∼200–2,000 MPa
Max. Cortical bone elastic modulus	24.5 GPa	24.2 GPa
Aspect ratio of plateau	1.46	1.52

Patient 2	Fractured (Left)	Unfractured (Right)
Trabecular bone elastic modulus	∼50–2,000 MPa	∼60–2,000 MPa
Max. Cortical bone elastic modulus	23.7 GPa	24.7 GPa
Aspect ratio of plateau	1.55	1.55

### Comparison of surgical risk factors

#### Patient 1

In patient 1, the tibial component in the fractured tibia was placed 0.74 mm more medial, 2.44 mm more distal, 1.73° more valgus and 18.83° more externally rotated compared to that in the unfractured tibia (see Supplementary Material for more details).

**FIGURE 3:**
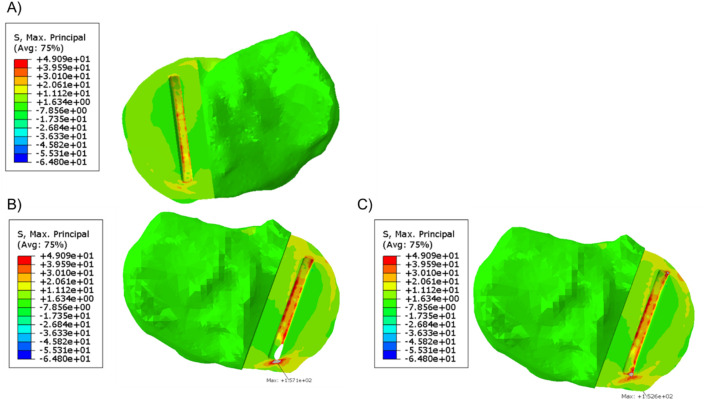
**(A)** Maximum principal stress plot of the unfractured tibia of patient 1 when a 1,000 N load was applied. **(B, C)** Maximum principal stress plot of the fractured tibia when a 1,000 N load was applied **(B)** when the keel slot cut through the tibia and made a hole and **(C)** when the keel slot was shifted backwards.

When a 1,000 N load was applied to the tibial component in the unfractured tibia, the highest maximum principal stress was 49.1 MPa at the anteromedial corner of the keel slot (Figure 3A). In the fractured tibia, the highest maximum principal stress was 157.1 MPa when the keel was placed in the middle of the keel slot in the anteroposterior direction which created a hole in the anterior cortex (Figure 3B). When the keel slot was moved backwards to avoid making a hole in the bone, the highest maximum principal stress was 152.6 MPa (Figure 3C). The highest stress was found at the anteromedial corner of the keel slot in both cases.

**FIGURE 4:**
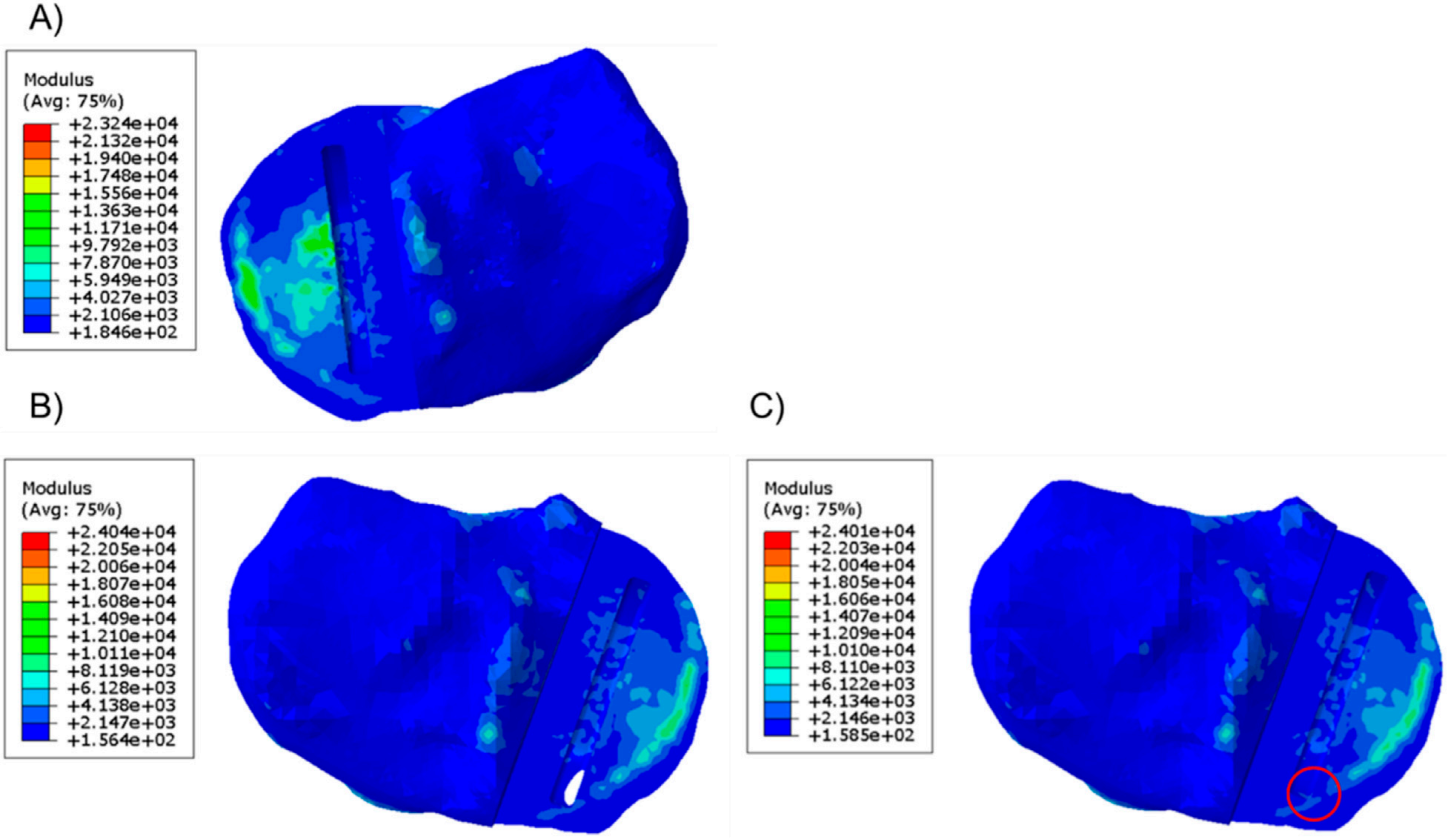
Material plot of the **(A)** unfractured and fractured tibia **(B)** when the keel slot cut through the tibia and made a hole and **(C)** when the keel slot was shifted backwards. The front of the keel slot cut through the cortical bone or cut into the cortical bone in the fractured tibia, marked by the red circle.

In the unfractured tibia, the keel slot was far away from the peripheral cortical bone while in the fractured tibia, no matter where the keel slot was made, the anterior cortex which is stiffer than the central trabecular bone was damaged (Figure 4).

**FIGURE 5:**
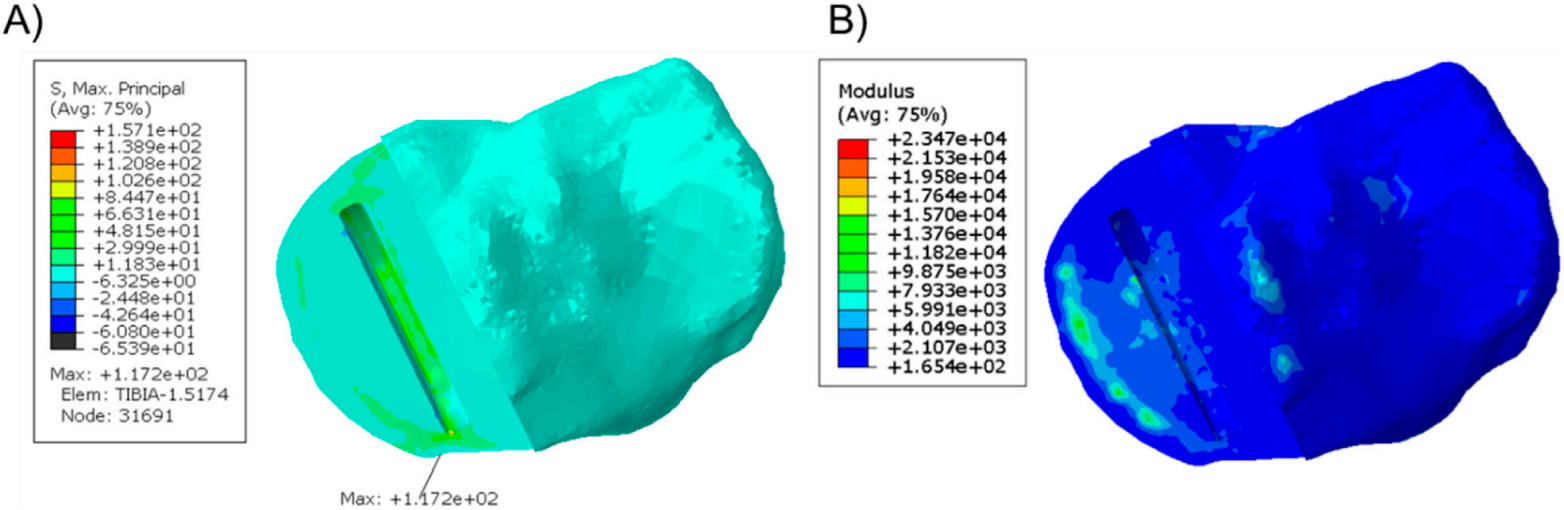
**(A)** Stress plot and **(B)** material plot of the unfractured tibia when the fractured cuts were replicated. The front of the keel slot was cut into the cortical bone.

When the fractured tibial resection was replicated in the unfractured tibia, the highest stress was 117.2 MPa at the anteromedial corner of the keel slot, which was 2.4 times the stress when the component was placed at its original unfractured position. The material plot showed that with this tibial resection, the keel slot would cut into the hard peripheral cortical bone regardless of the tibia it was made in (Figure 5).

**FIGURE 6:**
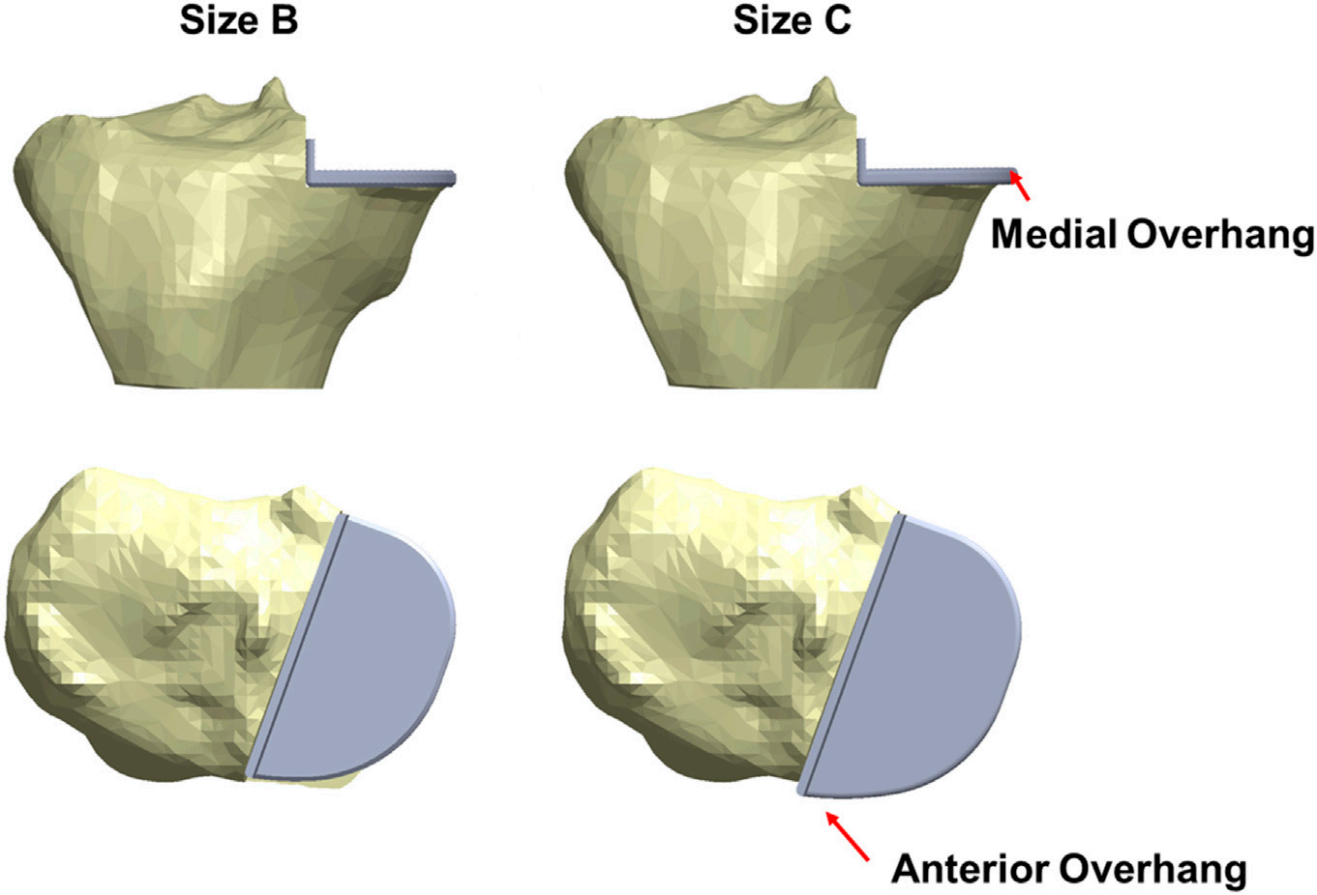
The fractured tibia of patient 1 with a Size B (left) or a Size C (right) tibial component implanted with the same vertical and horizontal tibial resections. A better coverage and less medial and anterior overhang are shown when a Size B component was implanted.

When a Size B component which is about 2 mm narrower and 3 mm shorter than a Size C component was implanted into the fractured tibia, good coverage of the bone was shown beneath the tray (Figure 6) and the maximum stress value was 62.0 MPa at the anteromedial corner of the keel slot.

#### Patient 2

**FIGURE 7:**
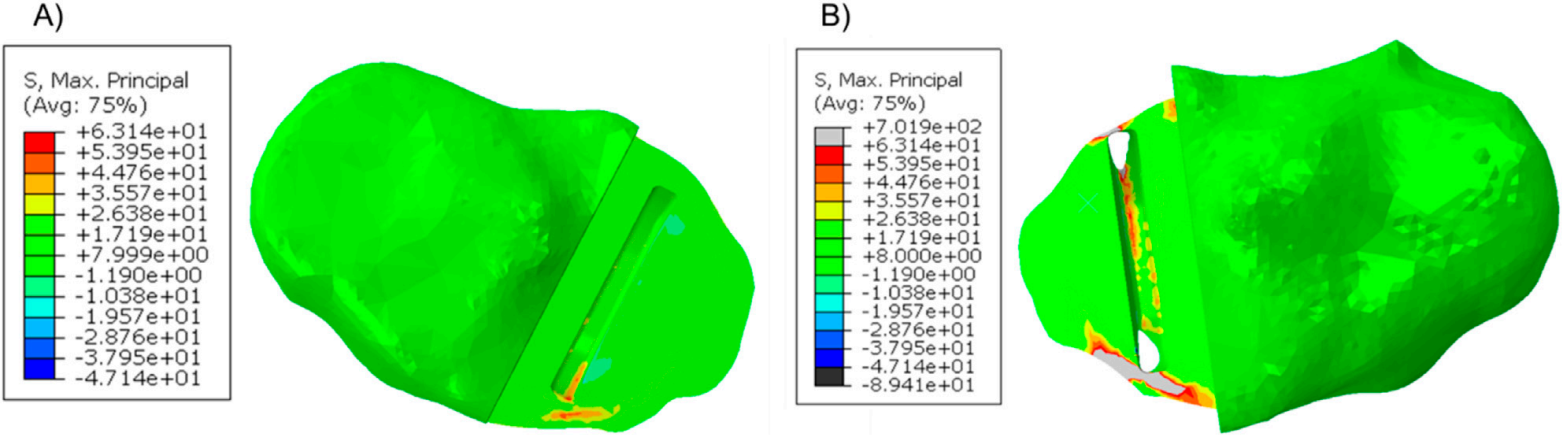
Maximum principal stress in the **(A)** unfractured and **(B)** fractured tibia of patient 2.

In patient 2, the tibial component in the fractured tibia was 3.95 mm more medial, 1.36 mm more distal, 1.47° more valgus and 16.40° more internally rotated (see Supplementary Material for more details). Holes were made while making the keel slot in both the anterior and posterior cortex. The highest maximum principal stress in the unfractured and fractured tibia was 63.1 MPa and 701.9 MPa, respectively (Figure 7).

**FIGURE 8:**
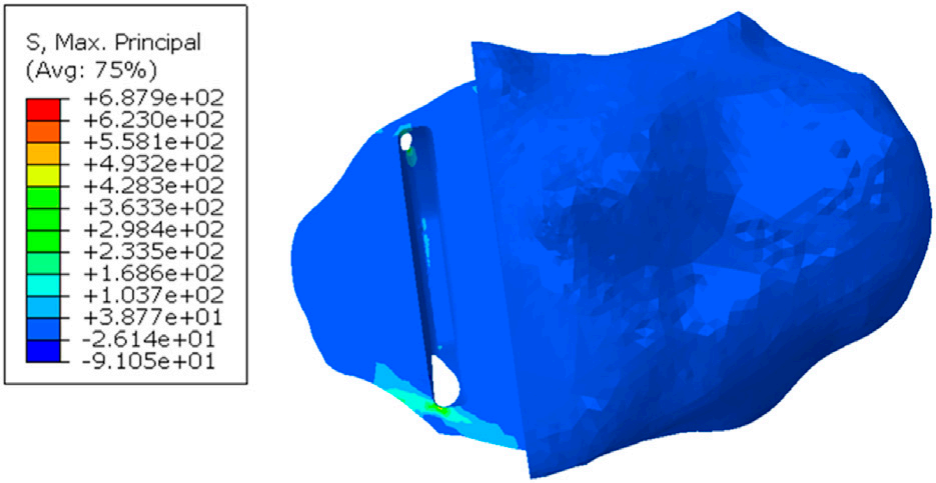
Maximum principal stress in the fractured tibia of patient 2 when a Size AA component was implanted and loaded.

When a Size AA component was implanted in the fractured tibia, the highest stress value was 687.9 MPa near the hole in the anterior cortex next to the keel slot (Figure 8).

**FIGURE 9:**
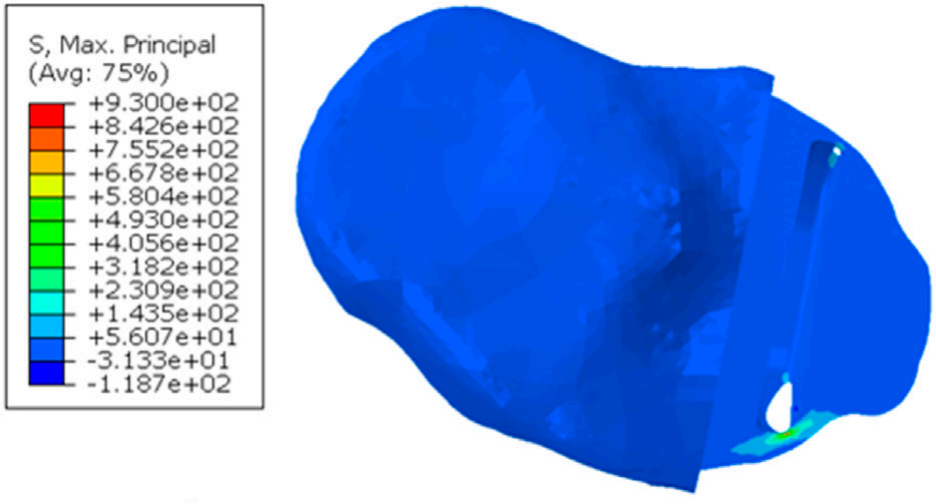
Maximum principal stress plot when the fractured cuts were replicated in the unfractured tibia of patient 2.

When the tibial resection in the fractured tibia was replicated in the unfractured tibia, the keel slot penetrated both the anterior and posterior cortex. The highest maximum principal stress was 930.0 MPa at the anteromedial corner of the keel slot near the hole (Figure 9).

### Effects of component’s position and orientation on risk of fracture

**FIGURE 10:**
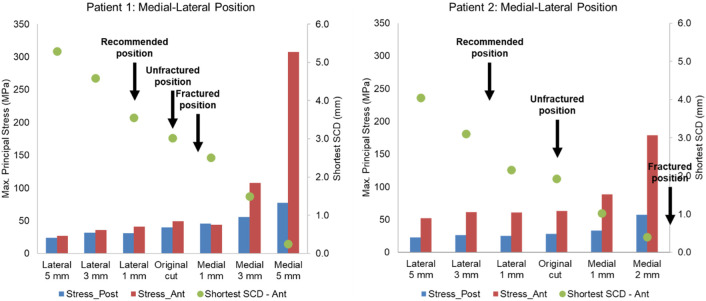
Highest maximum principal stress in the anterior and posterior part of the proximal tibia and the shortest anterior slot-cortex distance (SCD) in the unfractured tibia of patient 1 and patient 2 when the tibial component was translated in the mediolateral direction.

**FIGURE 11:**
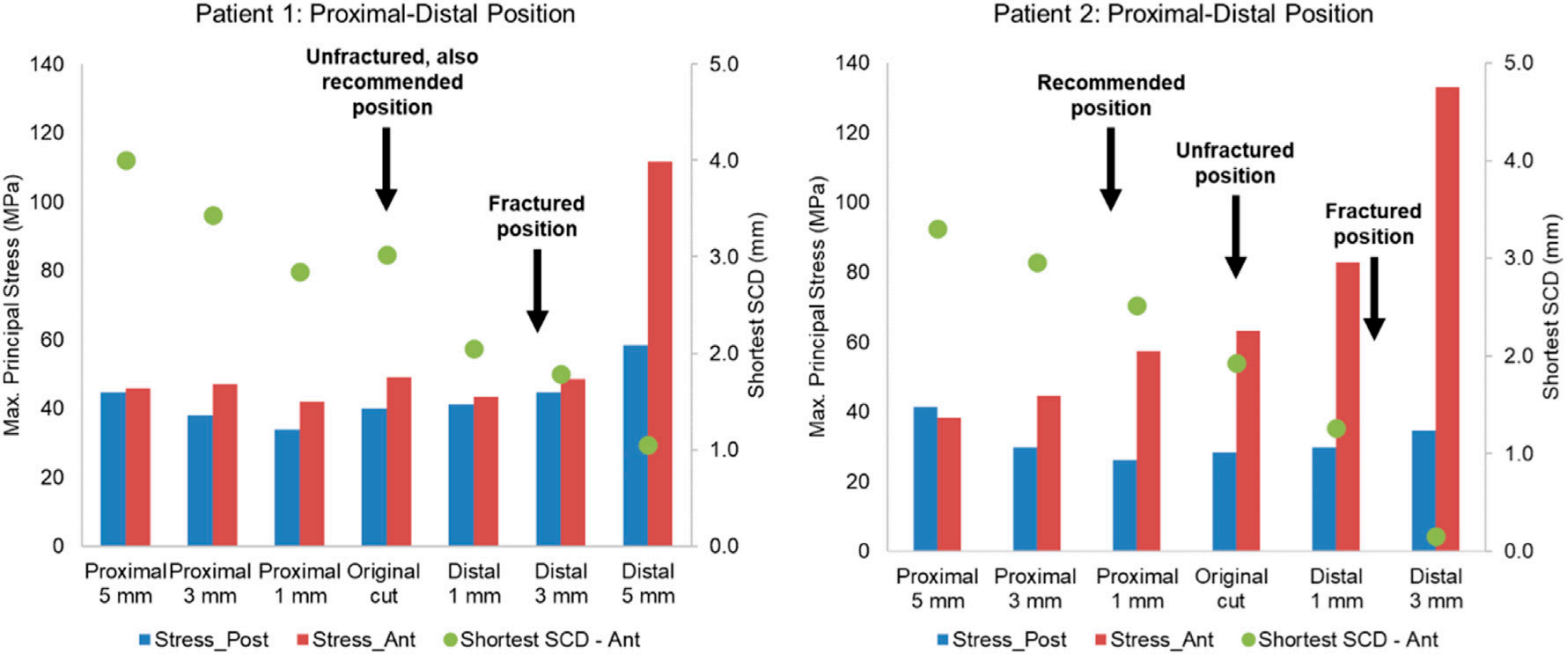
Highest maximum principal stress in the anterior and posterior part of the proximal tibia and the shortest anterior slot-cortex distance (SCD) in the unfractured tibia of patient 1 and patient 2 when the tibial component was translated in the proximal-distal direction.

**FIGURE 12:**
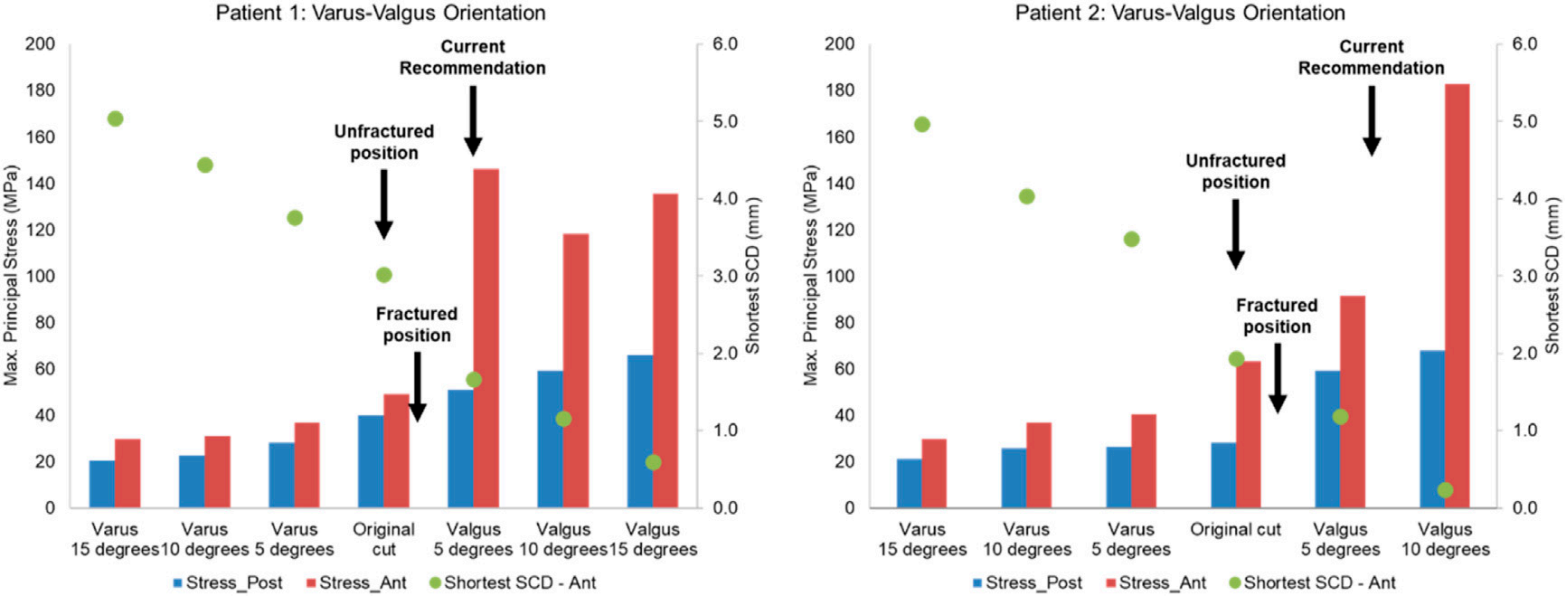
Highest maximum principal stress in the anterior (ant) and posterior (post) part of the proximal tibia and the shortest anterior slot-cortex distance (SCD) in the unfractured tibia of patient 1 and patient 2 when the tibial component was rotated in the varus-valgus direction.

The post-operative radiographs of patient 1 and patient 2 were assessed by a senior surgeon (DWM) and recommendations to the components’ mediolateral, proximal-distal and varus-valgus position based on the current surgical guidance were made (Figures 10, 11, 12).

**FIGURE 13:**
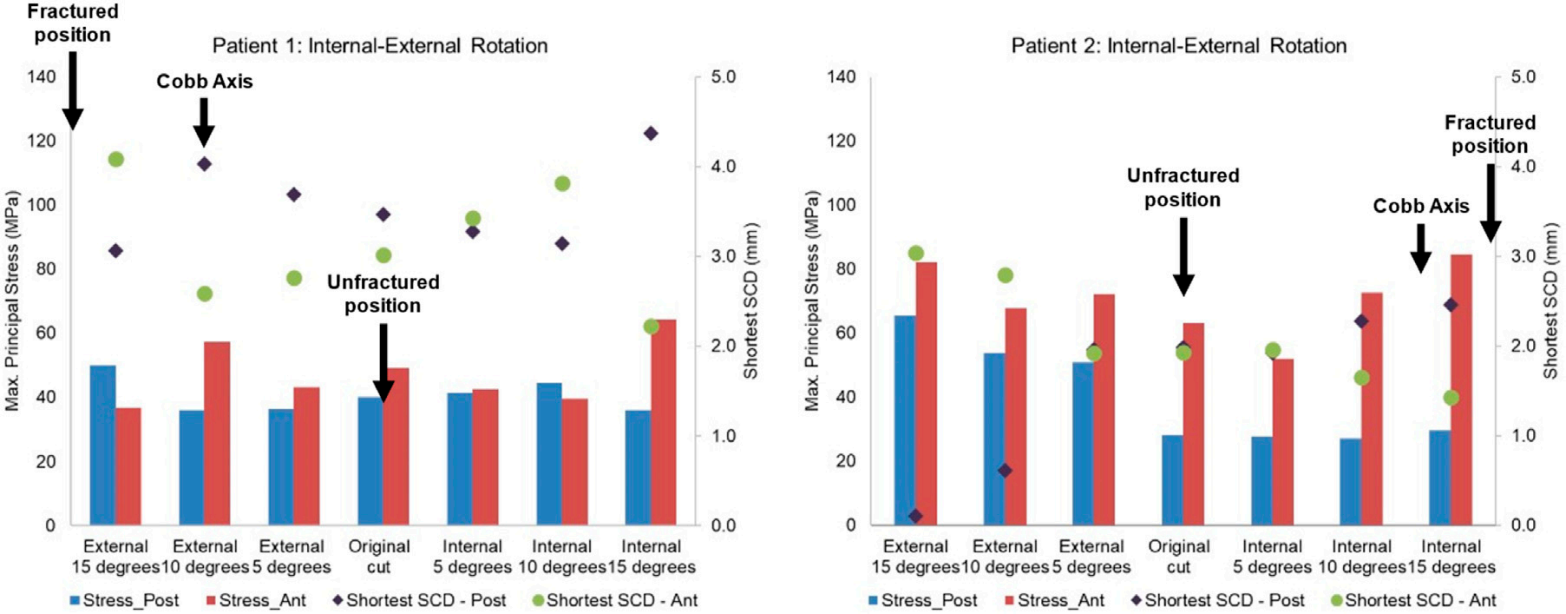
Highest maximum principal stress in the anterior and posterior part of the proximal tibia and the shortest anterior slot-cortex distance (SCD) in the unfractured tibia of patient 1 and patient 2 when the tibial component was rotated in the internal-external direction.

Lateral or proximal translation of the tibial component did not increase in the highest maximum principal stress in both unfractured tibias (Figures 10, 11). Rotating the components 5° into valgus significantly increased the highest stress in both patient 1 and patient 2, by 198% and 46% respectively (Figure 12). Two other damaging errors were making the vertical cut too medial and making the horizontal cut too distal. The stress doubled when the vertical cut was moved by 2 mm or more medially in both patients (Figure 10). A 60% increase in stress was seen when the horizontal cut was moved by 3 mm distally in patient 2 and it increased by almost 130% when the cut was moved by 5 mm distally in patient 1 (Figure 11). The increase in stress was within 35% when the component was rotated internally or externally by 5, 10 or 15°. Larger stress was shown at 10- to 15-degree rotation in either direction (Figure 13).

## Discussion

This study investigated the risk factors for TPF after OUKR by studying the knees of two high-risk patients who received bilateral OUKR but had a TPF in one knee and a good result in the other. The unfractured tibia of each patient acted as the comparator. It was found that in both patients, patient-related risk factors, such as bone quality, tibial size, and tibial plateau morphology were the same in the fractured and unfractured tibias. In contrast, surgical factors were very different in the fractured and unfractured tibia, and the differences were similar in both patients. This study therefore suggests that in high-risk patients the risk of fracture can be substantially reduced by optimally positioning components. The study has also identified the main errors that should be avoided in these patients. The margin for error in these high risk patients is very narrow, and much narrower than for normal patients.

Differences were found in the position and orientation of the tibial components in the fractured and unfractured tibias and the pattern was the same in both patients. In the fractured tibias, compared to the unfractured: the horizontal cut was more distal, the vertical cut was more medial, the components were more valgus and were more mal-rotated in the long axis. The FE analysis suggests that these individual errors may increase the maximum principal stress in the bone. However, when they occur together, as they did in both patients, the increased stress would be so large in these high-risk patients that it would be expected to cause a fracture. The FE results suggested that the errors increased the stress in the first patient by a factor of three (from 49 MPa to 157 MPa) and in the second patient, who was at higher risk, being smaller, by a factor of ten (from 63 MPa to 702 MPa).

To avoid the vertical tibial cut from being too far medial it should be positioned, as recommended, just medial to the apex of the medial tibial spine. The vertical cut of the two unfractured tibias, although near this position, could have been closer. The recommended direction of the vertical cut, when using the hanging leg position, is towards the anterior superior iliac spine. This direction is similar to the Cobb axis, an anatomical tibial axis defined by Cobb et al. (2008). It is difficult to precisely align the vertical cut, but this study has shown, from the fracture point of view, the direction is not critical with an error of 10°–15° being acceptable: in patient 1 and 2 respectively compared to the Cobb axis, the unfractured vertical cuts were 10° internally and 13° externally rotated, whereas fractured vertical cuts were 9° externally and 4° internally rotated. The horizontal cut should be as shallow as possible, so a 3 G-clamp should be used (Zimmer Biomet, 2019; Goodfellow et al., 2015). If the flexion gap is too tight, cartilage on the femoral side should be removed until the gap is large enough for the drill guide. The varus/valgus alignment is more controversial. It is recommended that this should be neutral. However, in both unfractured cases, the components were in 5° of varus and the FEA suggested that had they been neutral, a fracture probably would have occurred. Therefore, in high-risk cases, with marked tibia vara (MPTA = 80° in patient 1° and 79° in patient 2) the component should be implanted in about 5° of varus.

**FIGURE 14:**
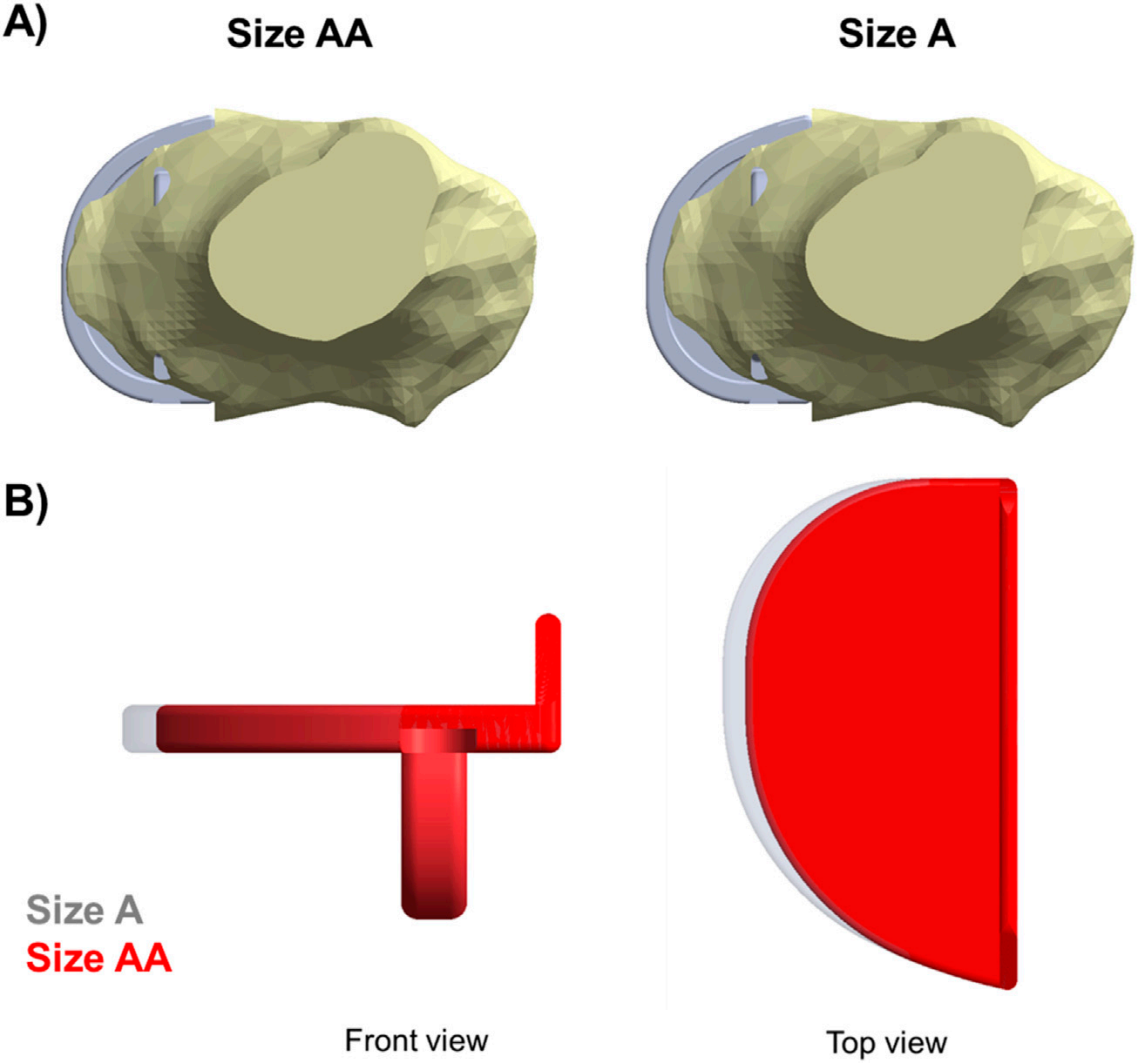
**(A)** Both Size A and Size AA components showed anterior and medial overhang in the fractured tibia of patient 2. **(B)** Comparison between a Size A (grey) and a Size AA (red) tibial component.

In the fractured tibia of patient 1, using a Size B component instead of a Size C reduced the highest maximum principal stress by around 60% (to 62 MPA), to a value only slightly larger than that in the unfractured side (50 MPa). Therefore, the fracture probably would have been avoided. The Size C component had both anterior and medial overhang. In contrast, the Size B component had no anterior overhang and minimal medial overhang, so was the optimal size component (Figure 6) (Zimmer Biomet, 2019). As the component is aligned with the posterior cortex, the shorter keel of the B is further from the front of the tibia, thus decreasing the high anterior stress and risk of fracture. In contrast, had the Size AA component been implanted instead of the A in the fractured tibia of patient 2, a fracture would still have occurred as there was no appreciable decrease in stress (688 MPa vs. 702 MPa). This is because the length of the keel and component is the same in AA and A, although AA is narrower (Figure 14). For both size AA and A there was significant anterior overhang.

As well as determining the maximum principal stresses, we also determined the distance from the slot for the knee to the cortex (SCD). This is a similar measurement to the KCD, which has been shown to be related to fracture (Watrinet et al., 2024). We found that if the SCD was 3 mm or more, the maximum stress was below the level at which a fracture would occur. The lower the SCD, the higher the risk of fracture. In patient 2, the slot cut through the cortex both anteriorly and posteriorly in the fractured side.

The study has some limitations: Firstly, the findings only relate to very high-risk patients who are very small and have marked tibia vara. In large patients without marked tibia vara fractures are rare and other factors may be more important such as the keel saw cutting through the posterior cortex in error. In these patients, surgeons should adhere to the standard recommendations and implant the tibial component in neutral, not varus. Secondly, a single load case was studied, which was the posterior-most position of the bearing during step-up motion. In reality, the bearing is mobile and moves back and forth while the knee flexes and extends and the load transferred to the tibial component varies accordingly. As this study investigates the causes of TPF, which is related to the highest stress induced in the bone, the load case applied in this study was intended to simulate the worst-case scenario. This is the posterior-most position of the bearing during step-up movements (Pegg et al., 2016), and it tends to occur in knee flexion, e.g., stair-climbing when the knee joint is under high loads (Kutzner et al., 2010). Thirdly, a cemented component was implanted in the fractured knee of patient 2. However, cement decreases the risk of fracture (Mohammad et al., 2020a; Burger et al., 2022). So, a fracture would also have occurred had it been cementless. Fourthly, the press-fit of the cementless keel was modelled by resolving the initial overclosure using an interference fit contact algorithm in Abaqus/Standard. As the focus of this study is on the comparison of the risk of fracture among four tibias with the OUKR component implanted with the same interference, the contact algorithm used was sufficient to provide information on the factors that affected fracture within a reasonable analysis time. Also, the 2 mm-slice thickness in the CT scans might not provide the most accurate reconstruction of the tibial geometry. However, while the thickness is 2 mm, the pixel size was 0.781 mm in the transverse plane, giving us a reasonably detailed geometry of the peripheral shape and material distribution inside the tibia. Lastly, the tibial bone was defined as a heterogeneous linear elastic material while real bone exhibits plastic behaviour (Ghouse et al., 2019). However, as the material definitions were consistent across models, this gave us enough insights into the effects of surgical cuts on the risk of TPF.

## Conclusion

This study investigates the risk factors of TPF that might have led to the difference in outcomes in two bilateral OUKR patients who were at very high risk of TPF. Both patients received the same-sized tibial components in both knees while one of them fractured and the other one had good results. Suboptimal positioning of the vertical and horizontal cuts was most likely to be the cause of fracture in these two patients. The results suggest that in small patients with marked tibia vara and overhanging tibial plateaus (extramedullary type), contrary to current recommendations, the tibial component should be placed in varus (around 5°). The vertical cut should abut the medial tibial spine. A 3 G-clamp should be used appropriately to achieve satisfactory resection depth. Extreme internal rotation or external rotation (more than 15° from the Cobb axis) that may compromise bony coverage beneath the tibial tray and lead to medial and anterior overhang should be avoided. This makes the tibia more sensitive to errors in the other three directions. Over-sized components increase the risk of fracture. Components should be aligned with the posterior cortex and should not overhang anteriorly.

## Data Availability

The original contributions presented in the study are included in the article/Supplementary Material, further inquiries can be directed to the corresponding author.
