# Supplementary material for: Surgical factors that contribute to tibial periprosthetic fracture after cementless Oxford Unicompartmental Knee Replacement: a finite element analysis
Source: Front Bioeng Biotechnol. 2025 Apr 4;13:1543792. doi: 10.3389/fbioe.2025.1543792 (PMC12006801; doi:10.3389/fbioe.2025.1543792)
Supplement: Supplementary file 1 [file DataSheet1.pdf]

# *Supplementary Material*

## 1 Supplementary Tables

### Table 1.1: Positions and orientations of tibial resections in patient 1.

| Patient 1 | Right Leg (Fractured) | Left Leg | Comparison |
|---|---|---|---|
| Posterior slope | 9.78° | 10.16° | Very small difference (fractured cut less posterior by 0.38°) |
| Internal-External Rotation | 8.77° External to the Cobb Axis | 10.06° Internal to the Cobb Axis | Fractured cut is more externally rotated by 18.83° |
| Medial-Lateral position | • Anterior Cut Point 9.36 mm medial<br>• Posterior Cut Point 16.86 mm medial<br>• Mid-point 13.11 mm medial to the Cobb Line | • Anterior Cut Point 16.72 mm medial<br>• Posterior Cut Point 8.02 mm medial<br>• Mid-point 12.37 mm medial to the Cobb Line | There is no large difference in the medial lateral position of the mid-point of the AP cut (fractured cut is 0.74 mm more medial). As the difference in external/internal rotation is large, the anterior cut point in the fractured tibia is 7.36 mm more lateral. The posterior cut point in the fractured tibia is 8.84mm more medial |
| Proximal-Distal position | 12.57 mm | 10.13 mm | Fractured cut 2.44 mm deeper |
| Varus-Valgus Rotation | 3.50° Varus | 5.23° Varus | Fractured cut 1.73° more valgus |

### Table 1.2: Positions and orientations of tibial resections in patient 2.

| Patient 2 | Right Leg | Left Leg (Fractured) | Comparison |
|---|---|---|---|
| Posterior slope | 8.53° | 7.32° | 1.21° less posteriorly sloped |
| Internal-External Rotation | 12.87° External to the Cobb Axis | 3.53° Internal to the Cobb Axis | Fractured cut is more internally rotated by 16.40° |
| Medial-Lateral position | • Anterior Cut Point 3.60 mm medial<br>• Posterior Cut Point 13.95 mm medial<br>• Mid-point 8.78 mm medial to the Cobb Axis | • Anterior Cut Point 14.05 mm medial<br>• Posterior Cut Point 11.41 mm medial<br>• Mid-point 12.73 mm medial to the Cobb Axis | Mid-point of the AP cut: fractured cut is 3.95 mm more medial. |
| Proximal-Distal position | 16.09 mm | 17.45 mm | Fractured cut 1.36 mm deeper |
| Varus-Valgus Rotation | 6.10° Varus | 4.63° Varus | Fractured cut 1.47° more valgus |
